# Inheritance of Chiari-Like Malformation: Can a Mixed Breeding Reduce the Risk of Syringomyelia?

**DOI:** 10.1371/journal.pone.0151280

**Published:** 2016-03-23

**Authors:** Susan P. Knowler, Henny v/d Berg, Angus McFadyen, Roberto M. La Ragione, Clare Rusbridge

**Affiliations:** 1 School of Veterinary Medicine, Faculty of Health & Medical Sciences, University of Surrey, Guildford, Surrey, United Kingdom; 2 Fitzpatrick Referrals, Eashing, Godalming, Surrey, United Kingdom; 3 akm-stats, Glasgow, Scotland, United Kingdom; Faculty of Animal Sciences and Food Engineering, University of São Paulo, BRAZIL

## Abstract

Canine Chiari-like malformation (CM) is a complex abnormality of the skull and craniocervical junction associated with miniaturization and brachycephaly which can result in the spinal cord disease syringomyelia (SM). This study investigated the inheritance of CM in a Griffon Bruxellois (GB) family and feasibility of crossbreeding a brachycephalic CM affected GB with a mesaticephalic normal Australian terrier and then backcrossing to produce individuals free of the malformation and regain GB breed characteristics. The study family cohort (n = 27) included five founder dogs from a previous baseline study of 155 GB which defined CM as a global malformation of the cranium and craniocervical junction with a shortened skull base and increased proximity of the cervical vertebrae to the skull. T1-weighted sagittal DICOM images of the brain and craniocervical junction were analysed for five significant traits (two angles, three lines) identified from the previous study and subsequent Qualitative Trait Loci analysis. Mean measurements for mixed breed, pure-breed and baseline study groups were compared. Results indicated that mixed breed traits posed less risk for CM and SM and were useful to distinguish the phenotype. Moreover on the MR images, the filial relationships displayed by the traits exhibited segregation and those presenting the greatest risk for CM appeared additive towards the severity of the condition. The external phenotypes revealed that by outcrossing breed types and with careful selection of appropriate conformation characteristics in the first generation, it is possible to regain the GB breed standard and reduce the degree of CM. The four GB affected with SM in the study all exhibited reduced caudal skull development compared to their relatives. The craniocervical traits may be useful for quantifying CM and assessing the possibility of SM thus assisting breeders with mate selection. However, such a system requires validation to ensure appropriateness for all breeds at risk.

## Introduction

Chiari-like malformation (CM) is a developmental abnormality involving the shortening of the entire skull base, reduced caudal cranial fossa volume and increased proximity of the cranial cervical vertebrae to the skull [1–4]. The consequential disturbance of parenchyma and cerebrospinal fluid (CSF) flow through the foramen magnum [5, 6] results in fluid-filled cavities in the spinal cord, a condition commonly known as syringomyelia (SM). Both disorders are known to be associated with clinical and behavioural signs of pain and other neurological deficits [7, 8] in dogs and humans [9, 10]. Numerous studies have revealed the complex nature of the CM and SM and the relationship between the two conditions [11–13] but little is known about their inheritance. The heritability of SM has been estimated in the Cavalier King Charles Spaniel (CKCS) as moderately high (h^2^ = 0.37 ± 0.15 standard error) [14] and prevalence increases with age [15–17]. CM is ubiquitous in the CKCS [18] and has an estimated prevalence of 65% in Griffon Bruxellois (GB) [19, 20]. One study investigating the breeding programme of CKCS (n = 550) and GB (n = 93) found 70% and 73% respectively of offspring were free of SM if their parents were SM clear over 5 years of age but when both parents were SM affected the risk of SM was 92% in CKCS and 100% GB [21].

### Genetic Basis of CM and SM

Pedigree analysis of human familial aggregations of Chiari type 1 malformation which is similar to CM, suggest both autosomal dominant with reduced penetrance [22–24] and autosomal recessive [25] but most likely the pattern of inheritance is oligogenetic and determined by the cumulative effect of variants in various genes. Canine CM and SM supports this multifactorial nature of inheritance [26–28]. Quantitative analysis of both CM and SM has been undertaken in the GB (n = 155) [1]. Six highly significant traits for CM and SM, confirmed by subsequent quantitative trait loci (QTL) analysis [29], were associated with five Canis Familiaris Autosomes (CFAs). One trait, angle FAC, was unique in that it was highly significant for CM but not for SM. Another, the diameter of a ‘best fit occipital lobe circle’ (f-diameter) is strongly associated with a genomic region on CFA2 containing a single candidate gene, *Sall-1*, [30] mutations of which are involved with branchial arch development and can be associated with Chiari type I malformation in human (Townes-Brocks syndrome) [31, 32]. These former studies provide the five baseline trait values for the current study.

### Crossbreeding

Crossbreeding, was defined by McGreevy and Nicholas [33] as a mating of individuals of different breeds that can result in ‘hybrid vigour’ or heterosis in the offspring and thereby alleviate inherited defects or inbreeding depression. They postulated that backcrossing the offspring of the cross with healthy individuals of the original breed can be a way of improving the health of the breed while preserving breed characteristics with less extreme phenotypes. The UK Kennel Club investigating inbreeding in 10 representative breeds found seven of them had lost over 90% of unique genetic variants in six generations [34]. Loss of genetic diversity could have serious implications for toy breed dogs predisposed to CM and SM. Breeding away from CM may prove difficult if the genes associated with this trait are ‘fixed’ in the population i.e. the variants of alleles associated with CM may be lost through genetic drift and no other available form [35]. Introducing new genes into the population by crossbreeding has been suggested as a means of reducing incidence of inherited diseases [36].

### Support for breeders

Dealing with an adult onset polygenetic condition like canine CM/SM is extremely problematical for breeders. In response to a request from the UK Cavalier Club, the veterinary profession has provided informal breeding guidelines for SM [37] and evidence suggests these measures have been successful in reducing prevalence [17] and effectiveness especially of earlier onset SM [21]. In 2012 the British Veterinary Association/Kennel Club (BVA/KC) launched a CM/SM Health Screening Scheme [38] as a means to standardizing the MRI protocol [38] and provide estimated breeding values (EBVs) to support their Mate Select services [39]. Although radiographs [19], computed tomography (CT) [40, 41] ultrasound [42] thermal imaging [43] and head conformation [44] have been used to indicate CM and risk of SM, magnetic resonance imaging (MRI) remains the most specific and sensitive means of diagnosis for these conditions and forms the basis of screening prior to breeding.

### Aims

1. To investigate the effectiveness of a crossbreeding a brachycephalic GB with CM and a mesaticephalic Australian terrier without CM as a means of reducing the incidence of traits associated with CM and risk for SM.
2. To elucidate the inheritance by comparing phenotypic traits related to both CM and SM that have been previously shown to be statistically significant [1] for the conditions.
3. To clarify the phenotype of CM with an ultimate aim of improving the CM/SM grading system and generate robust EBVs that are appropriate for all breeds.

## Materials and Methods

### Study cohort

The family (n = 27) comprised three foundation bitches C,D, H and two foundation dogs E and K that were part of the previously mentioned larger 155 GB cohort [1] used in genetic studies [29]. Furthermore, foundation Dog E was the offspring of a CM/SM affected GB that formed part of separate family (n = 32) where CM was investigated using radiographs [19]. The study cohort has two key black and tan coat GB siblings; a CM affected male (dog A) involved in the outcross to the Australian terrier bitch and his unaffected CM sister (bitch G) whose pure breed GB male offspring was used in a backcross to the F1 hybrid. Unlike the majority of countries, in the Netherlands (the breeder’s home), the black and tan coated GB is called a Griffon Belge and considered a separate breed (race) to the GB by the Federation Cynologique Internationale (FCI) [45]. However, in order to ease reading, different races of Griffon Bruxellois are referred collectively as GB in this manuscript. Two of the dogs in the extended family cohort (dogs A and H) had three matings each (i.e. comprising six matches) and the two older dogs that had previously been bred and scanned made a total of eight matches in the study.

Apart from Dog E, the entire family group were owned by co-author and breeder Hv/dB. Mating decisions were entirely those of Hv/dB and based on her assimilated knowledge of the CM and SM, the MRI status of the parents, head shape and coat colour. Selection for conformation in dog breeding is subjective and morphometric measurements were not considered by the breeder. Observations of head shape from previous matings that produced SM affected dogs prompted her selection of dogs with the longest skulls to mate with her CM affected dogs with shorter skulls. Selection was not based on head shape only, other factors were taken into consideration e.g. temperament and gait. All the dogs lived in the same household during the whole study period with similar vaccinations, exercise and raw meaty bones diet, thus minimising any environmental factors that might influence multifactorial traits such as CM and SM.

### Magnetic resonance imaging

T1-weighted sagittal DICOM images of the brain and cervical region were available for 26 of the 27 dogs. Dog 27 was euthanized when severe CM/SM was diagnosed in a preliminary MRI before a sagittal image was obtained. 19 offspring were imaged at 12–15 months. Seven dogs were rescanned and their revised CM and SM status reported. All the dogs in the study were scanned at the same veterinary centre using a 0.2 Tesla MRI machine (Esaote Grande, Italy) [46]. The protocol and grading of CM and SM were according to the BVA / KC CM/SM Health Scheme [38]. To ease reading, these are simplified: CM0 = normal, CM1 = intermediate, CM2 = affected. SM0 = normal, SM1 = intermediate, SM2 = affected. The MRI evaluations (grades) for CM and SM for the four F1 hybrids, their parents and two F2 backcross progeny were performed by the official BVA/KC CM/SM Health scheme [38]. The remaining dogs were graded by Netherlands KC grading scheme at Utrecht University using a system adopted from the BVA/KC scheme and protocols.

### Morphometric measurements used for quantitative analysis

**Fig 1:**
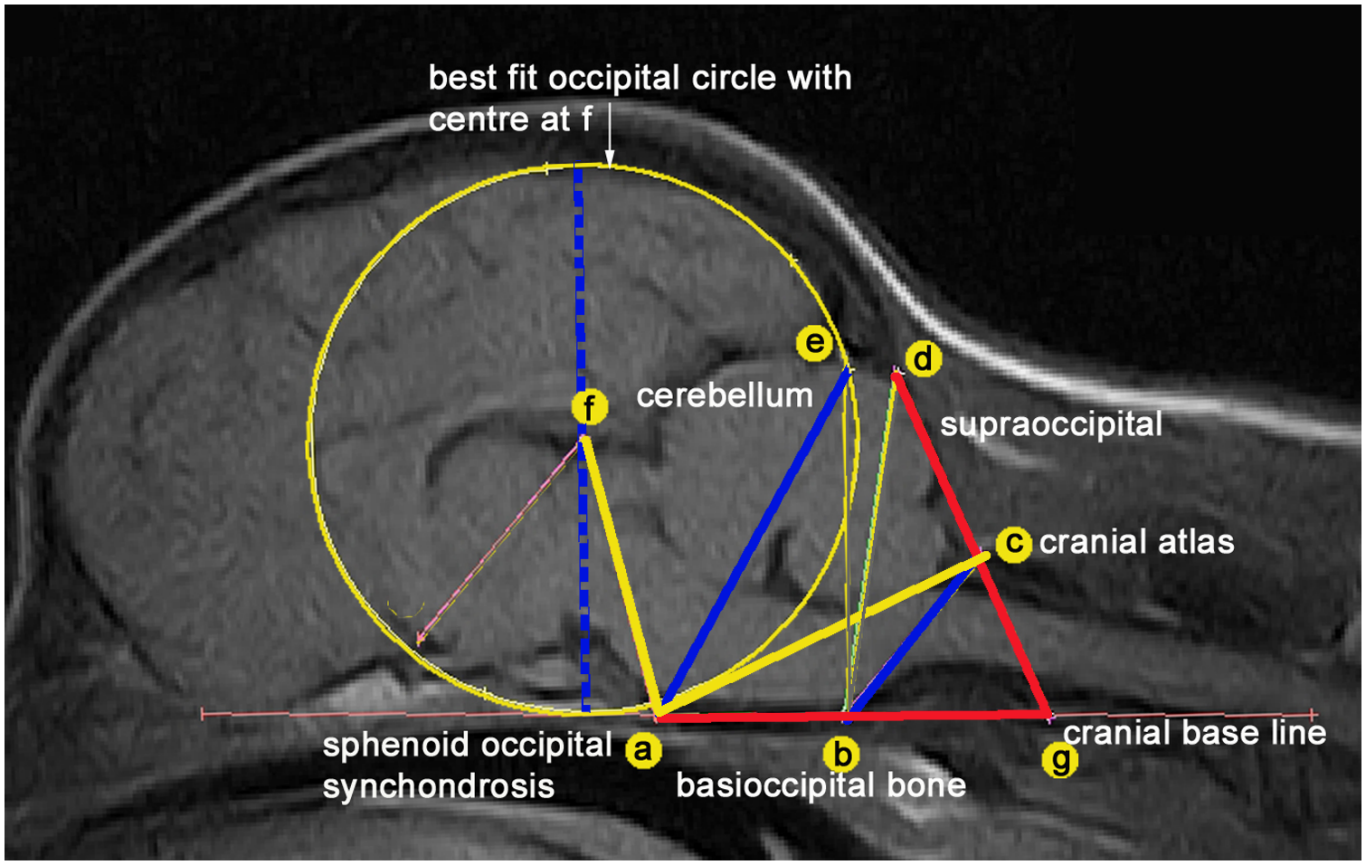
Midline sagittal T1-weighted MRI of brain and craniocervical region of a female GB backcross. A framework of measured lines and angles is used to assess conformational features associated with CM verified in a previous study [1]. (A) dorsum of spheno-occipital synchondrosis (B) basion of basioccipital bone (C) rostral edge of the dorsal lamina of the atlas (D) junction between the supraoccipital bone and the occipital crest (E) most dorsal point of intersection of the cerebellum with the occipital lobe circle (F) centre of occipital lobe circle placed on the extended cranial baseline (AB) (G) intersection point with the extended AB baseline and DC. The five traits measured in the study are lines ae, bc and f-diameter (blue) and angles FAC (yellow) and AGD (red).

Fig 1 illustrates the three lines ae, bc and f-diameter and two angles FAC and AGD that were significantly associated with CM and four of the five significant with SM in the previous baseline study (n = 155 GB) [1] selected for quantitative analysis.

Using the previously validated technique [1], the images were imported into Mimics 16.0 Materialise (Technologielaan 15 3001 Leuven Belgium). Adobe Photoshop 4 (http://www.adobe.com) was used to resize and aligned the framework of angles and lines and while keeping the ratios constant.

### Statistical analysis

Due to the lack of statistical independence within the family of dogs and the small samples no statistical testing was carried out. All measurements of traits were tabulated in MS Excel and imported into IBM SPSS^®^ (version 22). Descriptive data used boxplots to illustrate the range of variation within the CM and SM in a standardized way for distribution of data.

### Ethics Statement

This retrospective study was based on the analysis of images taken as part of routine clinical assessments. Ethics approval for this project was not sort because the dogs were family pets that lived with owner and breeder Hv/dB. All breeding, both mixed cross and pedigree, complied with the rules and authorization of the Raad van Beheer op Kynologisch Gebied. This is the principal cynological organization in the Netherlands which oversees pedigree dog health and welfare and regulates breeding registrations, including inspections of breeding premises, microchipping and DNA profiling. DICOM images were obtained for diagnostic purposes for determining CM and SM status and followed the protocol set out by the BVA [38].

## Results

### Study group CM/SM status and family relationships

**Fig 2:**
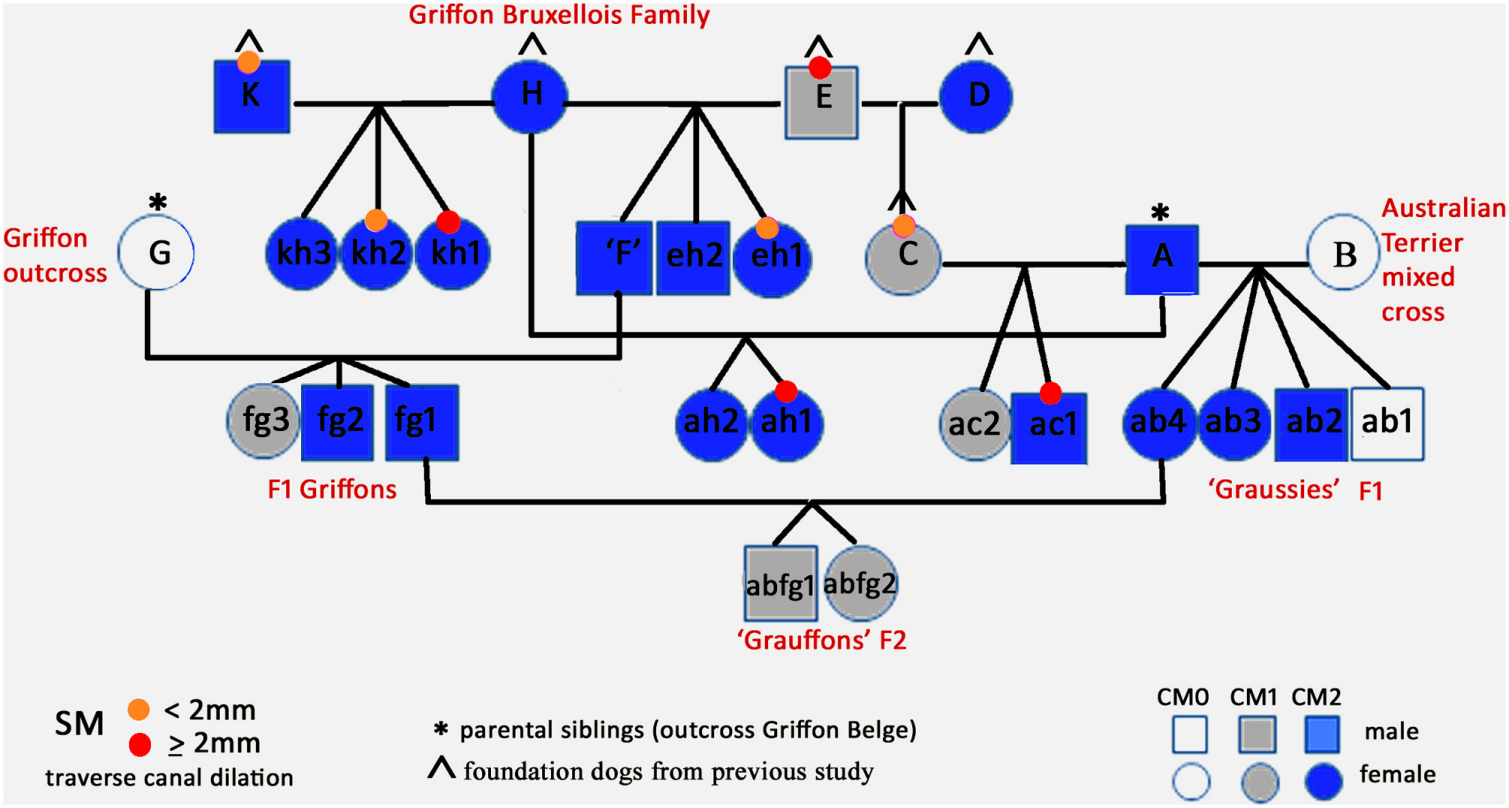
Study cohort GB family tree (n = 27) including a mixed cross to an Australian terrier and subsequent backcross. Offspring were designated the combined letters (lowercase) of their parents. Bitch C and Dog F were both a parent and an offspring so designated a single letter as a first generation parent (P1) to ease reading. Hence ed1 is renamed ‘C’ and ‘eh3’ renamed ‘F’.

The CM/SM status and family relationships are summarized in Fig 2 and in Table 1.

**Table 1:** Summary of CM and SM status in family study group.

Parental cross	Sire			progeny number				progeny number			Dam		
	CM/SM status			CM			litter	SM			CM/SM status		
	CM	SM	code	CM0	CM1	CM2	Total (N)	SM0c	SM1c	SM2c	code	CM	SM
AxB*	cm2	sm0b	**A**	1	0	3	**4**	3	1	0	**B**	cm0	sm0a
**AxC**	cm2	sm0b	**A**	0	1	1	**2**	1	0	1	**C**	cm1	sm1b
**AxH**	cm2	sm0b	**A**	0	0	2	**2**	1	0	1	**H**	cm2	sm0b
**ExH**	cm1	sm2b	**E**	0	0	3	**3**	2	1	0	**H**	cm2	sm0b
**KxH**	cm2	sm1c	**K**	0	0	2	**2**	0	1	1	**H**	cm2	sm0b
**ExD**	cm1	sm2b	**E**	0	1	0	**1**	0	1	0	**D**	cm2	sm0a
**FxG**	cm2	sm0c	**F**	0	1	2	**3**	3	0	0	**G**	cm0	sm0c
**ABxFG** #	cm2	sm0c	**FG1**	0	2	0	**2**	2	0	0	**AB4**	cm2	sm0c
**total (N) progeny**				**1**	**5**	**13**	**19**	**12**	**4**	**3**			

* Mixed cross

^#^ Back cross.

Offspring were designated the combined letters (lowercase) of their parents to facilitate understanding of the relationships. Bitch C and Dog F were both a parent and an offspring so designated a single letter as a first generation parent (P1) to ease reading. Hence ed1 is renamed ‘C’ and ‘eh3’ renamed ‘F’. Only one of the 19 offspring was CM0 (F1 hybrid ab1). There were four offspring with CM1; GB ac2 and fg3 and both second filial (F2) backcross progeny (abfg1 and abfg2). The 13 remaining offspring were CM2.

The progeny could not be confirmed lifetime clear of the disease because SM can be a late onset disease and the offspring were MRI screened at one year old. According to the BVA/KC nomenclature for SM, dogs screened less than three years of age are designated by the letter c. However, in this manuscript, to facilitate easier reading, a letter accompanying the SM grade is not stated in the text unless the dog underwent a MRI aged three to five years (designated b) or greater than five years (designated a). Three progeny (ac1, ah1 and kh1) and one parent E were SM2 affected. Two dogs, C and K, were SM1a and b respectively. Dogs eh1 and kh2 were both SM1 at one year but when re-scanned at three years eh1 remained SM1b. In contrast, F1 Hybrid ab3 was SM1 at one year and SM0 when rescanned at 2.7 years. Parents B and D were SM0a.

### Magnetic Resonance image morphometric measurements

**Fig 3:**
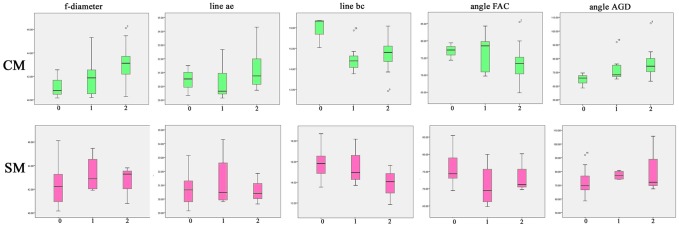
Boxplot distribution of significantly associated traits for CM and SM BVA/KC grades (n = 26). Top row; 0 = CM0 (normal), 1 = CM1 (intermediate), 2 = CM2 (affected). Bottom row; 0 = SM0 (normal), 1 = SM1 (intermediate), 2 = SM2 (affected).

S1 Table provides the five morphometric measurements made on T1 weighted sagittal available DICOM images for the family group (n = 26). Descriptive statistics for significant traits for both CM and SM are provided as Boxplots in Fig 3.

Table 2 compares the mean, maximum and minimum trait values for the six mixed breed offspring and the 12 pure-breed offspring relatives compared to previous baseline study (GB control cohort) together with the trend for CM and SM risk [1]. The means of the mixed breed group poses less risk for all traits than the other groups and the pure-bred group less risk than the control group.

**Table 2:** Descriptive Statistics for five traits in Mixed, Pure and control GB Groups.

		CM traits				
		*f-diameter*	*Line bc*	*Line ae*	*angle FAC*	*angle AGD*
Cohort	*value*	*mm*	*mm*	*mm*	*degrees*	*degrees*
**Mixed Breed Offspring n = 6**	**Mean**	**41.96**	**16.48**	**30.7**	**74.36**	**69.09**
	Minimum	40.24	15.34	29.14	70.44	65.13
	Maximum	45.31	18.69	32.67	79.4	74.35
**Purebred GB offspring n = 13**	**Mean**	**42.8**	**14.68**	**30.61**	**74.53**	**79.41**
	Minimum	40.2	11.85	29.27	67.63	63.52
	Maximum	46.11	16.84	33.02	84.31	105.7
**GB control group n = 155**	**Mean**	**42.47**	**13.15**	**31.68**	**71.2**	**81.93**
	Minimum	37.8	10.68	28.09	52.83	64.49
	Maximum	47.85	17.27	36.4	86.51	109.21
***CM and SM***	***less risk if***	***smaller***	***longer***	***shorter***	***wider***	***smaller***

**Fig 4:**
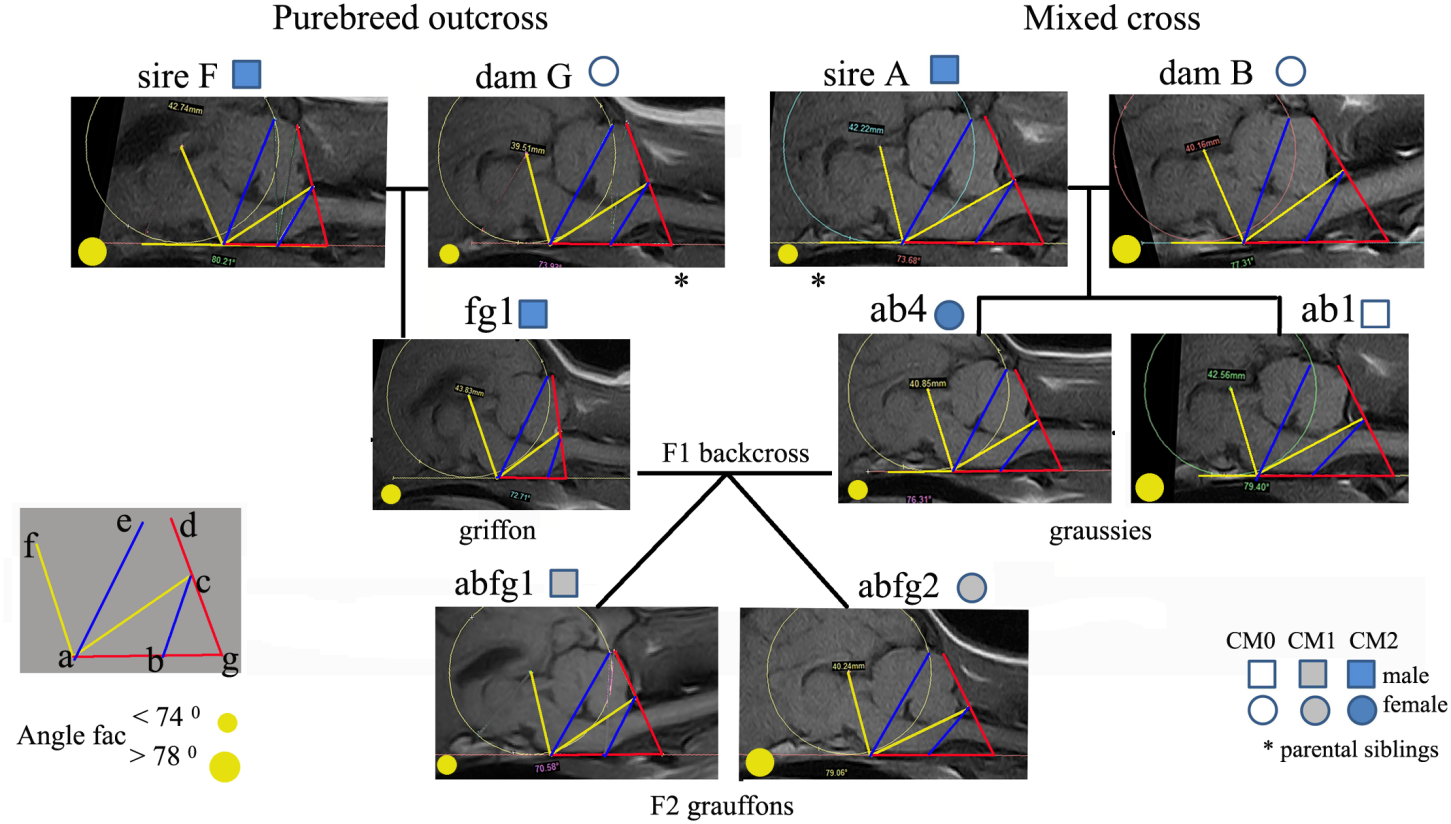
Key morphometric measurements made on the MRIs of dogs in three generation pedigree of the F2 Backcross. The CM0 F1 hybrid ab1 provides an additional control comparison. The lines and angles have been linked together providing a visual representation of the interrelationships between the individual traits. Sire fg1 and son abfg1 have similar values for angle FAC(~70°) but different values for line bc. Similarly, dam ab4 and daughter abfg2 have similar trait values for line bc (~15.7) but different values for angle FAC.

Table 3 provides individual values of the morphometric traits for the three generation F2 backcross arranged as a pedigree so that comparisons are easier to view. Fig 4 illustrates the traits as a framework superimposed on the MRIs.

**Table 3:** Morphometric measurements for three generation the F2 backcross pedigree.

Breed/cross	P1 GB	P1 GB	F1 GB	F2 backcross	F2 backcross	F1 Hybrid	P1 GM	P1 Australian T	
Dog code	F	G	fg1	abfg1	abfg2	ab4	A	B	*Less risk for CM
CM status	CM2	CM0	CM2	CM1	CM1	CM2	CM2	CM0	if value is
**f-diameter**	43.0	40.8	43.8	45.3	**40.2**	40.3	42.2	40.2	**smaller**
**line bc**	14.5	16.1	13.5	**17.7**	15.7	15.8	16.8	18.7	**longer**
**line ae**	30.2	30.5	31.0	32.7	**29.1**	29.7	32.1	29.3	**shorter**
**angle FAC**	80.2	73.9	69.7	70.6	**78.5**	72.9	73.7	80.0	**larger**
**angle AGD**	77.8	69.5	92.0	**65.1**	**66.3**	69.7	66.9	58.6	**smaller**

The degree of risk for CM is indicated by the trend in values (*final column of the Table 3). Both F2 backcross progeny exhibited varying degrees of intermediate CM1, but bitch abfg2 has only one out of the five risk factors: a shorter line bc. However dog abfg1 has three of the five risk factors; larger f-diameter, longer line ae and smaller angle FAC. The dogs with no CM (B and G) have a smaller f-diameter, longer line bc, short line ae and smaller angle AGD. Furthermore, Bitch G (CM0), when compared to sibling A with CM2, had a smaller f-diameter and line ae (less risk), similar line bc and angle FAC and angle AGD.

### Syringomyelia

**Fig 5:**
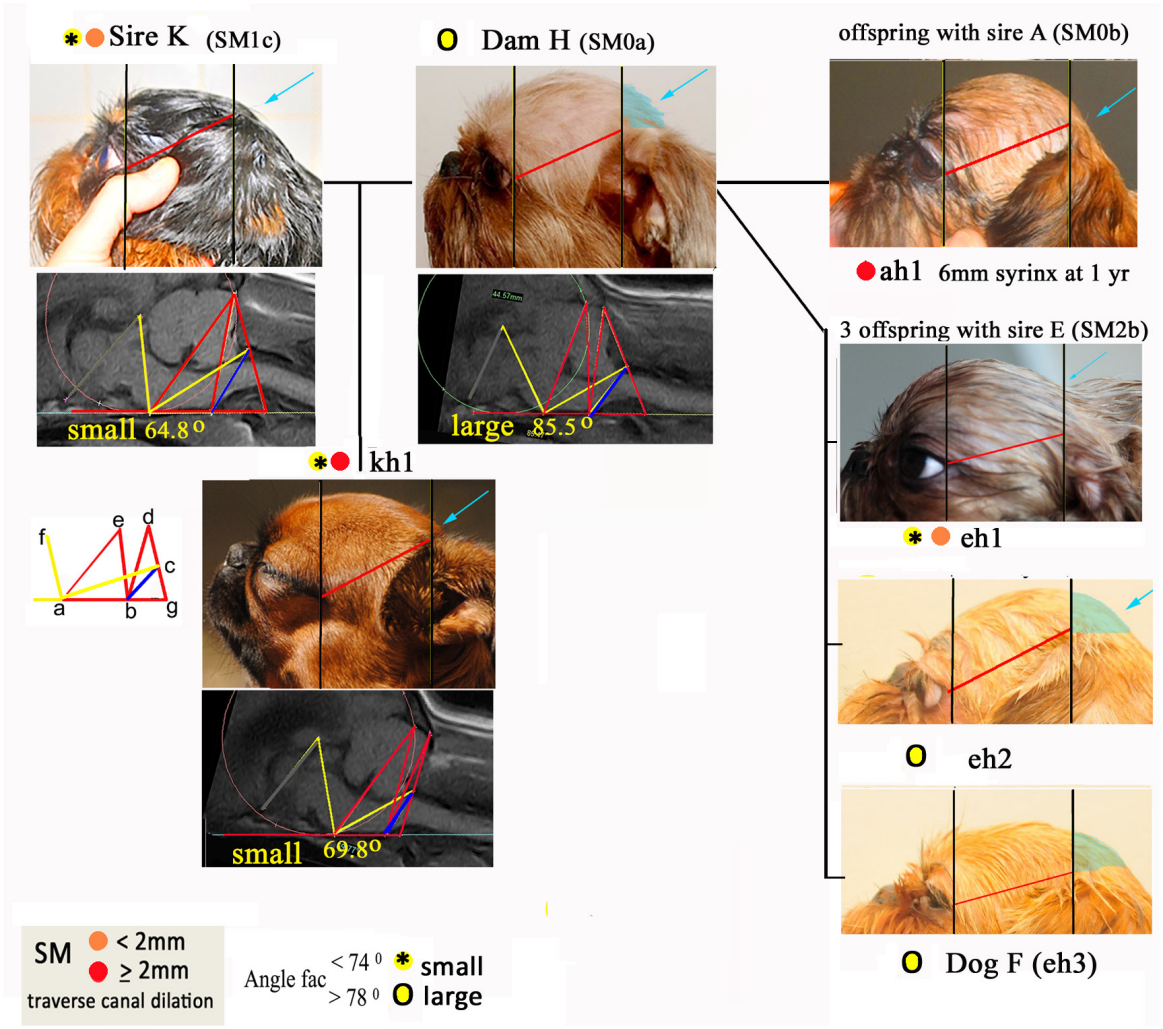
Head conformation and associated angle FAC in six relatives of Bitch H with and without SM. TW1 sagittal MRI of the caudal fossa and cranial-cervical junctions with superimposed morphometric framework of lines and angles for parents and offspring enhances comparison. The differences in the size of angle FAC are reflected in the lack of skull development caudal to the ear pinna (behind the ears) for dogs with SM compared to dogs with no SM (‘normal’ caudal skull shaded aqua colour). The photos of the heads have been resized to allow comparison using two vertical lines (black) placed at the outer eye and the origin of the external pinna (red) a consistent distance apart.

Four of the dogs in the cohort (E, ac1, ah1 and kh1) had syringomyelia (SM2) and four dogs (K, C, eh1 and kh2) had central canal dilation less than 2mm (SM1). All these dogs are related to either Dog E with SM2 or Dog K with SM1 (Fig 1) with the exception of offspring ah1. Angle FAC was of special interest because it had been found to be significant for CM and not SM in the former baseline study [1]. Fig 5 illustrates Bitch H with 5 offspring from three different sires. This bitch (CM2 SM0a) was described by the owner/co-author as having a ‘huge head’ compared to the breed average and she has a large angle FAC. When mated to Dog K with small angle FAC (SM1b) the two offspring with small angle FAC (kh1 and kh2) both had SM. The offspring with larger angle FAC (kh3) did not have SM. Fig 5 the head shape and MRIs of Dogs K,H and kh1 are illustrated in together with further examples of Bitch H’s other matches with Dogs A and E. The SM affected dogs demonstrate the lack of skull development caudal to the ear pinna (behind the ears) compared to dogs with no SM. This supports the radiographic evidence in the previous GB family study [19] and head conformation in the CKCS [44].

### External Phenotype

**Fig 6:**
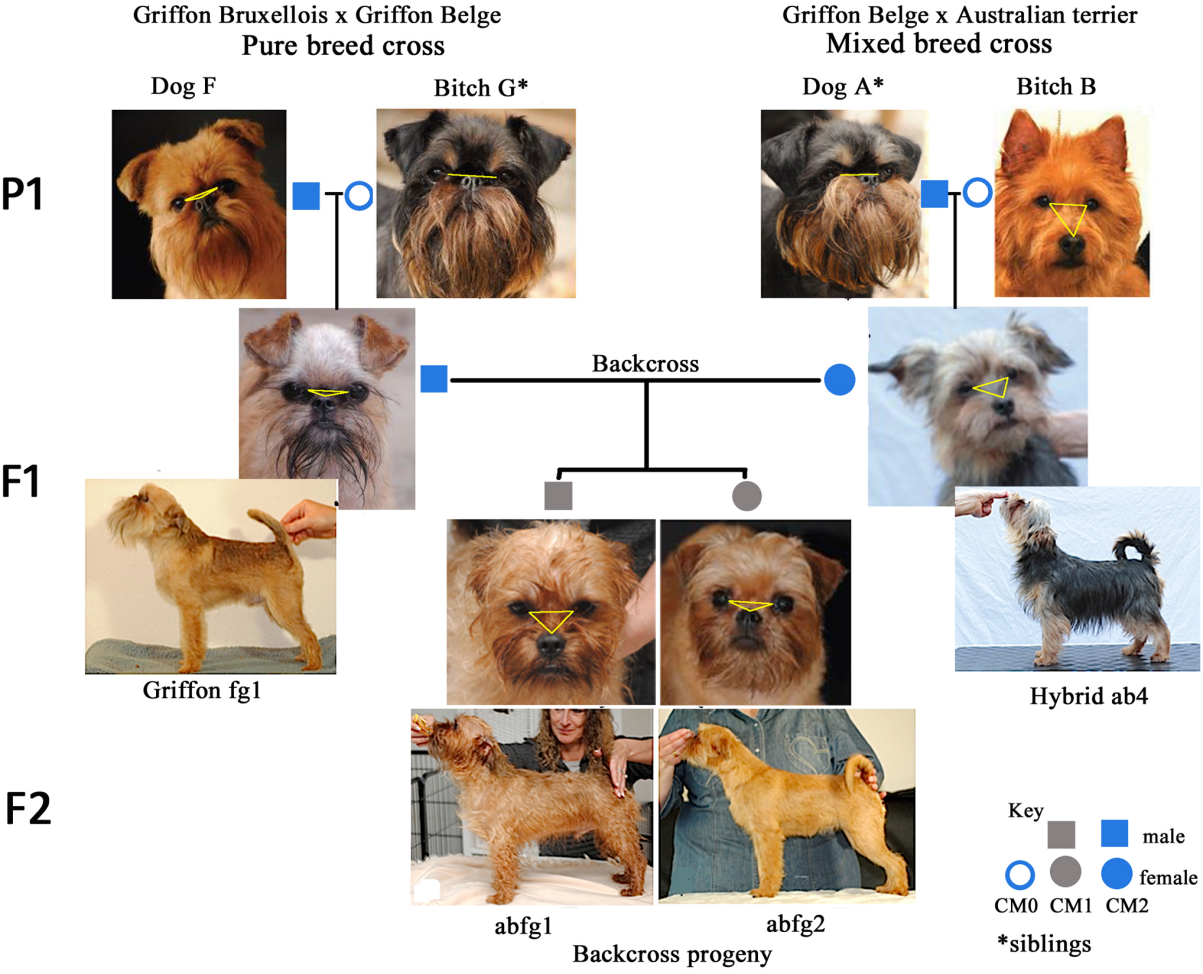
Facial features of three generation pedigree of F2 Backcross and F1 and F2 body conformation. P1 = first parents F1 = first filial generation F2 = second filial generation. P1: Australian terrier (Dam B) has a longer muzzle which is well below eyes (yellow triangle) and upright ear pinna then the brachycephalic GB. Sire A has greater palpebral aperture so the eyes appear proportionally larger to the face. F1 Hybrid: ab4 eyes are “smaller” (more orbital coverage) than the GB with muzzle and ear placement and body conformation intermediate between parents and incorrect for the GB.

The monkey-like face of the GB is considered one of the most desirable and therefore important features of the breed. The GB FCI and USA Breed standard dictates that the eyes” line up” horizontally with the nose, encouraging extreme brachycephaly and mid facial hypoplasia (S2 Table). Fig 6 depicts the head conformation characteristics of the three generations involved in mixed cross, GB outcross and subsequent GB backcross and body conformation of the F1 and F2 generation. It should be noted that breeders use the term brachycephalic (and used in this context) to infer the short length of the nose with respect to the skull and not to the cephalic index (ratio of the width and length of the skull excluding the nose).

All four P1 (brachycephalic GB dog F, siblings A and G and chondrodystrophic and mesaticephalic Australian terrier B are all FCI Breed Champions and therefore acknowledged distinguished examples of their breed type. As expected for complex traits involved in body shape and hair-coat, all F1 hybrids (ab) showed intermediate forms of the parents. They had relatively longer muzzel with a less pronounced undershot jaw than their brachycephalic GB sire (A). In the F2 backcross generation, male abfg1 has both head and body conformation faults for the Breed standard with a wider head than GB, more pronounced ‘stop’ and longer muzzle than his sibling. The eyeball has normal orbital coverage. By comparison F2 backcross abfg 2 most resembles the GB breed standard with the nasal planum level with the lower eyelid (flattened yellow triangle) and reduced orbital coverage resulting in the characteristic “large” eyes of the GB. The ear and body conformation is “set” correctly for a GB (similarities with purebred GB sire fg1).

## Discussion

CM has been shown to be a risk factor for SM and this developmental malformation can also be painful and result in decreased quality of life [18, 47]. In this study five conformation traits for CM were analysed in an extended family involving a mixed breed cross which took advantage of an accidental mating between a mesaticephalic breed and the brachycephalic GB inspiring the three year study. T1-weighted sagittal DICOM images were compared with a purebred outcross family with varying affectedness for CM and SM. It builds on data from four previous studies in the GB that investigates the phenotype, the risk of CM and SM and its inheritance and the successful identification of two Qualitative Trait Loci and candidate genes [1, 19, 21, 29]. Furthermore, analysis of familial morphometric differences in the eight litters and 19 progeny, we were able to identify potential traits that might be useful for grading the severity of CM and SM.

### Did the outcross reduce the risk of inherited CM and SM?

#### Chiari-like Malformation

An outcross does have the potential to reduce prevalence of inherited CM and SM. In eight matches only one offspring (hybrid ab1) was CM0. However his external conformation resembled his mesaticephalic Australian terrier dam and least desirable phenotypically. The purebred GB outcross with parent G with CM0 produced one of three offspring with CM1. Three remaining CM1 dogs were all related (E, C and ac2) suggesting they had inherited similar skull conformation. The 13 CM2 progeny all had at least one CM2 parent. However an exception was the F2 backcross progeny. These had less severe CM1 than both their CM2 parents.

#### Syringomyelia

Although the offspring were too young to confirm SM clear status, the morphometric measurements used in this study provide an indicator of risk for SM. For example, any reduction in distance between point B and C represents less area for the hindbrain. An increased f-diameter (height of the rostral cranial cavity) is a significant risk factor for CM and SM [1]. This increase in height is thought to be a developmental compensation to accommodate the forebrain and occurs in response to cranial base craniosynostosis and overcrowding in other parts of the skull [48]. In a previous study of 93 Griffons [1], 67 of the dogs had CM (72%) but nine of the 26 CM free dogs had SM and were similar to Dog E in this study. Although the cerebellum is not compressed or herniated into the foramen magnum, it is invaginated rostrally under the parenchyma of the occipital lobes and/or there is generalized ventriculomegaly indicating obstructed CSF dynamics. This is possibly due to arachnoid adhesions [12] but the possibility that there may be a thoracic or lumber syrinx cannot be ruled out because only the caudal spinal cord was imaged [49].

### Did the study indicate any patterns of inheritance for CM/SM?

This study is too small to make conclusions about inheritance. However, the F2 backcross had CM1 status from CM2 parents despite the high incidence of inherited CM in the other family members (Fig 2). These results, together with the fact CM is known to skip a generation in an earlier familial study of 33 GB [19], suggests the involvement of recessive traits that are protective against CM and/or gene penetrance is variable involving individual traits that are additive in severity. Line bc is associated with CFA9 and CFA24 and line ae with CFA14 [29], i.e. these segregated traits may or may not be expressed in any one individual as illustrated by the variations in F1and F2 generations.

SM2 dog E and SM1 dogs C and K were mated to SM0 dogs. Of their eight offspring two were SM2 and two SM1. SM0 parents A and H when mated together produced both an SM0 and SM2 offspring. All these findings support evidence from two previous studies of inheritance in the BG [19, 21].

### Is it possible to retain Breed Standard in an outcross?

Purebred dog breeding is considered an art not a science. Breed standards are descriptive guidelines for the appearance, movement and temperament of a dog decided by Breed Clubs and can vary from one country to another. Used as a tool for assessing a dog in the show ring, the interpretation of breed standard is subjective. The head and body conformation of the F2 backcross abfg is sufficiently similar to the GB breed standard that, in the next generation GB backcross (CM0), a suitable F3 offspring would be accepted in the GB Stud book of the European FCI. Since facial length has a different morphogenesis and embryological origin to that of the basisphenoid and occipital bones [50] it should be possible to select against a reduced hind skull. Author Hv/dB breeds for health and prefers the UK GB breed standard [51] that stipulates a muzzle longer than 1.5cm and is less extreme than the European FCI and American GB standard [52]. There is concern that the breed standard may encourage health problems related to conformation, for example, S2 Table compares the UK and American KC GB Breed Standard with the dysmorphic features of Crouzon or branchial arch syndrome in humans where there is abnormal development of the first branchial arch resulting in craniosynostosis and abnormal development of the eye sockets and mid-face [12, 53, 54].

The introduction of new genes in a mixed breed cross means progeny may be phenotypically less predictable. Despite this, the dog skull is particularly variable [55], and this study has illustrated that with careful selection of external and internal traits in the proposed mate, it might be possible to avoid risk of CM and SM and regain the external conformation which best characterises the breed. Details of how this was achieved can be found at http://www.cmsmtrust.org/index.php. Author and breeder Hv/dB provides an in depth graphic account of the mixed cross and pedigree breeding programme which explains her reasoning.

### Did the study help to clarify the CM phenotype?

The framework of traits proved useful in refining the definition of CM. Both the “angle” traits relate to the displacement of the atlas bone relative to the dorsum of the supraoccipital bone and skull base. An increased angle AGD reduces the volume (space) available for CSF to flow freely. Differences in angle FAC reflect the alignment of the atlas and supraoccipital crest, the degree of ‘invagination’ of the cerebellum under the cerebral parenchyma and proximity to the sphenoid occipital synchondrosis. Therefore, if line bc from the basion of the basioccipital bone to the rostral edge of the dorsal lamina of the atlas (i.e. distance across the craniocervical junction) is considered in combination with the other traits, this may impart an additional degree of risk for overcrowding and result in SM or painful CM. Since all the traits exhibited continuous variation, it is feasible that angle FAC, significant for CM, may be protective for SM when it is wide and that the combined effect on the caudal fossa volume might be additive if the angles and lines are considered together rather than individually.

This study supports the hypothesis that CM in the dog is a more global skull disorder rather than a caudal skull abnormality. Furthermore, the occipital bone insufficiency associated with rostral cranium doming seen in dogs K, kh1 and ah1 Fig 5 (aqua arrow) has been documented in other studies [18, 19, 56] and determined to be risk factor for SM in a study of conformation in the CKCS [44]. Repeat MRI screening for late onset SM offers the opportunity to monitoring morphometric changes over time [57].

### Support for breeders and health screening

The BVA/KC Health Scheme grading of CM based on the shape of the cerebellum and its degree of deformation does not take into account the recent research findings for GB [1, 20] and CKCS [13, 58, 59]. As yet there is no way to convert such findings into a simple objective measure that can predict CM and SM or means to distinguish a dog that will remain asymptomatic versus a dog that will develop pain and /or SM. Although the grading of SM objectively measures changes in the spinal cord central canal and/or syrinx transverse diameter, any minor central canal dilatation may not be readily demonstrated on low field MRI, especially if the operator technique is suboptimal. We suggest that a quantitative ‘scoring’ system is developed for CM and SM which incorporates craniocervical traits to provide a more objective grading for these conditions for breeding programs and EBVs. The Kennel Club (UK) have stated that they would consider favourably proposals from a breed club to outcross to address a health problem, particularly if based on scientific advice however an effective program would require a critical mass in terms of numbers of dogs and backing from the breed club i.e. is less likely to be successful if undertaken by an individual [60].

### Limitations of this study

This study was not a scientific experiment but part of a breeding program with much loved pets. It took advantage of an accidental mating between two different breeds. The data is limited but has all been provided by the goodwill of the owner Hv/dB. The expense and effort of maintaining so many dogs was considerable and the puppies that were rehomed as pets were not available for screening as originally planned. MRI screening prior to breeding does not require images of the entire brain so that measurements of the forebrain were not available. Since offspring were screened at one year, this was a prohibitive factor investigating SM which can be late onset.

Although the study sample size was small it includes all three races of GB (red rough coat), Petit Brabancon (red smooth) and Griffon Belge (Black and Tan rough coat). Furthermore, the family is part of a worldwide GB pedigree database of over 300 dogs with confirmed CM and SM status, with known relatives in Europe, Australia and the USA.

Mating decisions were entirely those of breeder and co-author Hv/dB based on 40 years’ experience and as a Dog Show Judge for 24 years. Such a program has to take into consideration not only other health issues (eyes heart, patella luxation, etc.) but the availability of suitable breeding dogs. An outcross with another breed is not supported by any GB Breed Clubs or the Kennel Club (UK). The financial cost for screening litters over a relatively short period of two and a half years, limited the total number of puppies that could be included in the study, despite additional funding provided by public donations (Syringomyelia DNA Research).

Finding suitable CM free dogs in breeds with a very small gene-pools and high prevalence of CM can be difficult for GB breeders and the reason why the F1 backcross was not mated to a CM normal dog. Furthermore, overuse of the limited MRI screened dogs shown to be clear of CM and SM (popular sire syndrome) would further reduce the gene pool [61, 62]. A mixed breed outcross has the advantage of reducing inbreeding depression and disease incidence [63].

## Conclusions

This is study of an outcross between a normal mesaticephalic Australian terrier and GB with CM and subsequent backcross to a GB. Techniques developed in an earlier study to quantify CM and SM in GBs were used as a control and applied to the extended family group to investigate inheritance of CM and SM in pure and cross breeding. Comparing the familial inheritance of five significant traits on MR images associated with CM and SM, we showed variants exhibited segregation and suggested that a protective role existed. The traits were useful to quantify CM and SM and to distinguish the phenotype. Furthermore such variants may be additive towards the severity of CM and SM. The definition of CM is refined as a more global cranium and craniocervical junction abnormally characterized by insufficiency of the supra and basioccipital bones with compensatory rostral cranium doming, shortening of the skull base and increased proximity of the cervical vertebrae to the occiput resulting in overcrowding of the neural parenchyma in the caudal fossa.

The external phenotype showed that by outcrossing and careful selection of appropriate conformation characteristics in the first generation, it is possible to regain the GB breed standard and reduce the degree of CM. The four dogs affected with SM in the study all exhibited reduced caudal skull development compared to their relatives. These craniocervical traits may be useful to quantify CM and risk of SM to assist breeders with mate selection. Such a system requires validation to ensure appropriateness for all breeds at risk.

## Supporting Information

S1 Table Five morphometric measurements made on T1 weighted sagittal available DICOM images for the family group (n = 26). (ZIP)

S2 Table UK and USA GB Breed Standard for head compared to facial dysmorphic characteristics of Crouzon Syndrome. [53] Craniosynostosis is characterized by premature closure of calvarial, cranial base, orbit and maxillary complex sutures. * Following concerns about the welfare impact of the conformation of some pedigree dogs many breed standards were modified by the Kennel Club (UK) so as not to encourage features that might prevent a dog from breathing, walking and seeing freely. Typically these changes were the insertion of downplaying words such as “slightly”, relatively” and “moderately.
(XLSX)
